# Draft Genome Sequences of *Lactobacillales* Isolated from the International Space Station

**DOI:** 10.1128/MRA.00942-20

**Published:** 2020-09-24

**Authors:** Achintya R. Bharadwaj, Nitin K. Singh, Jason M. Wood, Marilyne Debieu, Niamh B. O’Hara, Fathi Karouia, Christopher E. Mason, Kasthuri Venkateswaran

**Affiliations:** a Biotechnology and Planetary Protection Group, Jet Propulsion Laboratory, California Institute of Technology, Pasadena, California, USA; b Biotia, New York, New York, USA; c Department Cell Biology/College of Medicine, SUNY Downstate Health Sciences University, Brooklyn, New York, USA; d Blue Marble Space Institute of Science, Exobiology Branch, NASA Ames Research Center, Moffett Field, California, USA; e Space Research Within Reach, San Francisco, California, USA; f The WorldQuant Initiative for Quantitative Prediction, Weill Cornell Medicine, New York, New York, USA; University of Maryland School of Medicine

## Abstract

Nineteen strains from the order *Lactobacillales* were isolated from the International Space Station and commercial resupply vehicle, and whole-genome sequences (WGS) were generated. WGS would permit the characterization of these potentially pathogenic bacteria that have been adapting to the extreme conditions of the space environment.

## ANNOUNCEMENT

The order *Lactobacillales* consists of Gram stain-positive, facultative anaerobes validly described by Ludwig et al. (1). Members of the genus *Enterococcus* are found to possess human pathogenicity characteristics such as antibiotic resistance (2) and therefore pose health concerns for those on Earth and astronauts residing in the International Space Station (ISS). However, Aerococcus urinaeequi, a nonpathogenic strain, was first isolated from horse urine (3). Astronauts on long flights are immunocompromised due to microgravity-induced physiological and mental stress. Decreased immune response allows bacteria to take growth advantage due to their adaptability potential in the space environment (4). Understanding the genomic makeup of these potential pathogens will help the development of suitable countermeasure and mitigation strategies. Members of the order *Lactobacillales* isolated from the ISS and the commercial resupply vehicle (CRV) surfaces were Enterococcus faecalis, Enterococcus faecium, and Aerococcus urinaeequi (5, 6). E. faecalis and E. faecium have been reported as nosocomial isolates harboring vancomycin and ampicillin resistance (5). *A. urinaeequi* was isolated from a chronic kidney disease patient and has also been reported to be resistant to vancomycin (6). Further characterization of the whole-genome sequences (WGS) of these ISS environmental strains, including virulence genes, and subsequent confirmation in animal models are required to decipher their potential pathogenicity.

The strains used for the WGS were collected from three different ISS locations across two flights and seven different surface locations, including one field control on CRV6, and are detailed in Table 1 (7). The samples collected from the ISS were brought back to Earth and aseptically processed, and suitable aliquots of the sample concentrate (100 μl) were plated onto Reasoner’s 2A (R2A) or Trypticase soy agar (TSA) medium and incubated at 25°C for 7 days. A single well-isolated colony on a culture plate was archived at −80°C. Genomic DNA was extracted from the overnight-grown cultures on TSA medium using a ZymoBIOMICS DNA MagBead kit according to the manufacturer’s instructions.

**TABLE 1:** Metadata and genome statistics of *Aerococcus* and *Enterococcus* strains isolated from various ISS and CRV6 environmental surfaces during the Microbial Tracking-1 Flight Project^*a*^

Sample name	ANI (%)^*b*^	GenBank accession no.	Raw sequence accession no.	Flight no./location^*c*^	Location description	No. of contigs	Genome size (bp)	*N*_50_ (bp)	Median sequencing depth (×)	No. of QC reads	No. of raw reads	G+C content (%)
151250015-1-258-55	96(A)	JACGAN000000000	SRR12341118	F1-1	Cupola	35	1,981,406	130,552	282.59	6,701,742	3,361,020	39.5
151250015-2-258-56	96(A)	JACGAM000000000	SRR12341117	F1-2	WHC	36	1,981,307	130,552	645.54	15,523,394	7,803,035	39.5
151250009-4-258-51	96(A)	JACGAO000000000	SRR12341119	F1-4	Dining table	38	1,981,891	130,552	885.27	20,960,030	10,516,330	39.5
IIF2*SW-B2	99(B)	JACDPC000000000	SRR12341307	F2-2	WHC	26	2,928,643	679,975	736.61	22,911,864	11,494,983	37.4
IIF2SG-B4	99(B)	JACDPE000000000	SRR12341300	CRV6-2	Outside capsule	29	2,926,313	293,834	499.55	15,063,826	7,552,409	37.4
IIF3SG-B2	99(B)	JACDPF000000000	SRR12341299	CRV6-3	Outside capsule	20	2,948,392	1,487,444	600.00	19,513,428	9,781,100	37.3
IIF4SG-B3	99(B)	JACDPG000000000	SRR12341298	CRV6-4	Inside capsule	24	2,928,137	352,081	559.82	17,637,974	8,849,109	37.4
IIF4SG-B5	99(B)	JACDPH000000000	SRR12341297	CRV6-4	Inside capsule	22	2,929,029	680,116	675.00	21,186,826	10,633,808	37.4
IIF5SG-B2	99(B)	JACDPI000000000	SRR12341296	CRV6-5	Inside capsule	27	2,926,858	293,439	467.41	13,779,006	6,902,599	37.4
IIF6SG-B1	99(B)	JACDPJ000000000	SRR12341295	CRV6-6	Inside capsule	21	2,928,522	680,116	673.66	20,619,150	10,336,467	37.4
IIF6SG-B2	99(B)	JACDPK000000000	SRR12341294	CRV6-6	Inside capsule	23	2,928,581	352,365	835.71	25,542,378	12,807,968	37.4
IIF6SG-B4	99(B)	JACDPL000000000	SRR12341293	CRV6-6	Inside capsule	21	2,928,384	679,976	811.61	24,973,062	12,536,216	37.4
IIF7SG-B2	99(B)	JACDPM000000000	SRR12341305	CRV6-7	Inside capsule	19	2,948,759	1,487,531	595.98	19,817,626	9,946,584	37.3
IIF7SG-B3	99(B)	JACDPN000000000	SRR12341304	CRV6-7	Inside capsule	21	2,928,555	680,118	523.66	16,480,536	8,263,734	37.4
IIF8SG-B1	99(B)	JACDPO000000000	SRR12341303	CRV6-8	Inside capsule	20	2,948,399	1,487,531	543.75	17,797,982	8,925,021	37.3
IIF8SG-B2	99(B)	JACDPP000000000	SRR12341302	CRV6-8	Inside capsule	30	2,926,820	293,834	570.54	16,894,654	8,447,267	37.4
IIF8SG-B3	99(B)	JACDPQ000000000	SRR12341301	CRV6-8	Inside capsule	20	2,948,924	1,487,531	495.54	16,544,212	8,303,048	37.3
IIFCSG-B3	99(B)	JACDPD000000000	SRR12341306	CRV6-FC	Field control	30	2,926,028	293,439	570.54	17,408,136	8,727,751	37.4
IIFCSG-B5	95(C)	JACGAP000000000	SRR12341224	CRV6-FC	Field control	71	2,821,574	91,275	866.52	28,642,870	14,378,989	38.0

a Abbreviations: ANI, average nucleotide identity; F1, ISS flight 1; F2, ISS flight 2; WHC, waste and hygiene compartment; FC, field control (a sampling wipe was exposed to the air for 120 s at the center of CRV6); QC, quality control.

b The 16S rRNA gene sequences were retrieved from the WGS, and BLAST analysis was conducted against type strains of all 16S rRNA sequences in the NCBI database. The bacterial species identity was determined when the queried sequence showed >97.5% similarity with the 16S rRNA gene sequences of the type strain. The WGS of the nearest neighbor was further selected for ANI evaluation: A, *A. urinaeequi* DSM 20341^T^; B, E. faecalis DSM 20478^T^; C, E. faecium DSM 20477^T^.

c Hyphenated designations indicate the flight number followed by the location; for example, F1-1 indicates flight 1 and location 1.

The WGS of 19 bacterial isolates were prepared using the Illumina Nextera Flex protocol for library preparation, as used in similar studies (8). The NovaSeq 6000 S4 flow cell paired-end 2 × 150-bp platform was used to execute paired-end sequencing. FastQC v0.11.7 was used to validate the quality of the raw sequencing data (9). Adapter trimming and quality filtering were carried out using the software fastp v0.20.0 to perform quality control (10). The cleaned sequences were assembled using SPAdes v3.11.1 (11). The *N*_50_ values, numbers of contigs, and total genome lengths were generated using QUAST v5.0.2 and used to assess the quality of the final assembly (12). The average nucleotide identity was calculated by comparing all strains with their respective type strains, and their taxonomic affiliations, as well as genome statistics, are given in Table 1 (13). The NCBI Prokaryotic Genome Annotation Pipeline v4.12 was used for genome annotation. Default parameters were used for all software.

### Data availability.

This WGS project has been deposited at DDBJ/ENA/GenBank, and the accession numbers are given in Table 1 (BioProject accession no. PRJNA645454 with 16 strains and PRJNA649272 with 3 strains). The versions described in this paper are the first versions.
